# Assessing Job Satisfaction and Stress among Pharmacists in Cyprus

**DOI:** 10.3390/pharmacy10040089

**Published:** 2022-07-25

**Authors:** Georgios Stavrou, Olga Ch. Siskou, Michael A. Talias, Petros Galanis

**Affiliations:** 1 Ministry of Health, Nicosia 1448, Cyprus; 2 Health Policy and Planning Postgraduate Program, Faculty of Economics and Management, Open University Cyprus, Nicosia 2220, Cyprus; 3 Department of Tourism Studies, University of Piraeus, 18534 Pireas, Greece; olsiskou@unipi.gr; 4 Centre for Health Services Management and Evaluation, Faculty of Nursing, National and Kapodistrian University of Athens, 15772 Athens, Greece; 5 Healthcare Management Postgraduate Program, Open University Cyprus, Nicosia 2220, Cyprus; michael.talias@ouc.ac.cy; 6 Clinical Epidemiology Laboratory, National and Kapodistrian University of Athens, 15772 Athens, Greece; pegalan@nurs.uoa.gr

**Keywords:** job satisfaction, work stress, pharmacists

## Abstract

*Background*: Inadequate staffing, increased responsibilities and a high workload are some of the factors that are directly related to stress levels experienced by pharmacists, which in turn affect job satisfaction. *Objective*: The aim of this study was to assess job satisfaction and stress levels of pharmacists in Cyprus, involving those working in the public and private sector. *Materials and Methods*: A cross-sectional study was performed which involved the completion of the Job Satisfaction Survey (JSS) questionnaire to estimate job satisfaction, and the Perceived Stress Scale-14 (PSS-14) questionnaire to evaluate perceived stress. Data collection took place between January and March 2020 and the participation rate was 71.6% (*n* = 585). *Results*: Employees in private pharmacies overall reported higher levels of job satisfaction compared to public sector pharmacists. Public sector pharmacists were found to have stronger self-efficacy beliefs compared to other groups (*p* < 0.001). Female pharmacists had a higher average level of perceived helplessness than male pharmacists (*p* = 0.001). Regarding public sector pharmacists, it was generally observed that pharmacists working under the management of the Ministry of Health (MoH) had reduced job satisfaction than those working for other organizations. Additionally, pharmacists working under the management of the State Health Services Organization (SHSO) had the least overall perceived stress levels (*p* = 0.008), high self-efficacy beliefs (*p* = 0.006) and low perceived helplessness (*p* = 0.031) compared to pharmacists in other workplaces. Employees of private pharmacies were found to have higher levels of job satisfaction (*p* < 0.001) than SHSO pharmacists. However, those employees demonstrated increased perceived stress levels (*p* < 0.001) in comparison with SHSO pharmacists. *Conclusions*: Pharmacists’ job satisfaction is negatively correlated with perceived stress levels and helplessness, and positively correlated with self-efficacy beliefs. In the public sector, it seems that a re-evaluation is critical regarding the determinants that adversely influence job satisfaction amongst pharmacists.

## 1. Introduction

The fast pace and demands of modern life lead to stress in daily life [1]. Stress is a psychological and physical condition that manifests when a person is unable to cope successfully with demands and difficulties [2]. Emotions such as anger, anxiety, grief, despair, depression, and disappointment are characteristics of stress [3]. Job satisfaction is the individual’s emotional reaction toward their work [4,5,6]. Job satisfaction is usually calculated as an assessment of job characteristics combined with internal or external comparison standards [7,8,9].

There is great interest in the international literature regarding the assessment of job satisfaction in pharmacists and their stress in professional environments (private or public pharmacy, hospital environment, etc.). In addition, there is literature interest regarding their professional career/advancement and their intention to stay or abandon their profession [10,11,12,13,14,15]. The most common factors directly related to pharmacists’ stress are workflow interruptions and inadequate staffing [16,17]. Furthermore, increased responsibilities and greater workloads, insufficient workspace, working conditions, lack of guidelines by managers at the appropriate time and in general the existence of organizational problems and conflicts cause stress and even lead pharmacists to exhaustion (burnout) [18,19,20,21,22]. High stress levels in the pharmacy field often result from poor communication, unreasonable goals, and the promotion of a culture of long hours [23]. The body for pharmacists and in general the field of pharmacy in Great Britain (Royal Pharmaceutical Society of Great Britain) states that the public seems to have a high level of confidence in the knowledge, skills, and professional judgment (scientific knowledge) of pharmacists [24].

Studies in the USA have shown that the levels of job satisfaction and stress vary according to the role and responsibilities of pharmacists [25]. Several studies report relatively high job satisfaction among pharmacists, with male pharmacists slightly less satisfied than female pharmacists [10,26]. Community pharmacists seem to have higher levels of stress and state that they are less satisfied with their profession than hospital pharmacists and pharmacists in primary health care [10,27,28]. This phenomenon is likely attributed to the different roles of a community pharmacist.

## 2. Aims of This Study

This study aims to assess job satisfaction and stress of pharmacists in Cyprus, of both the wider public sector [Pharmaceutical Services (PhS), Health Insurance Organization (HIO), State Health Services Organization (SHSO), Ministry of Health (MoH)] and the private sector, specifically in private pharmacies (either as owners or employees). This group of pharmacists has been chosen because:

(a) They constitute the majority of pharmacists in Cyprus, since a very small percentage work in pharmaceutical companies, private hospitals, etc. (b) The implementation of the General Healthcare System (GHS) has brought many significant changes regarding the functioning and sustainability of private pharmacies (1st phase was implemented on 1 June 2019). In the wider public sector, changes have been made, mainly due to the implementation of the GHS, which have greatly influenced the daily lives of pharmacists (change in duties, transfer to another city). Following an announcement of the MoH dated 30 August 2019, 19 public pharmacies terminated their operations from 2 September 2019, in the context of the start of the first phase of the GHS (decision of the Council of Ministers, dated 6 May 2019, no. of decision 87.428).

In 2012, prior to the implementation of the GHS, in Cyprus, there were 8 public pharmacies in hospitals, 43 public pharmacies in health centers and 435 private pharmacies employing over 500 pharmacists [29]. Prior to the implementation of the GHS, private sector pharmacies served approximately 15% of the pharmaceutical market. Therefore, the largest share of the pharmaceutical market (85%) was served by public sector pharmacies (public hospitals, rural and urban health centers), since approximately 85% of the Cypriot population were eligible to receive pharmaceutical care from the public sector either free or almost free of charge.

The GHS compensates for the necessary medicinal products, which according to the relevant law can only be dispensed with a doctor’s prescription and are included in the Catalogue of Medicinal Products [30]. With the implementation of the GHS, beneficiaries can now receive their medication from all the private pharmacies contracted with HIO by submitting a prescription of a physician or a dentist also contracted with HIO. Some medicines, due to the provision of specialized pharmaceutical services, safety and/or the need to maintain records, are available exclusively from specific hospital pharmacies.

As a result, a large volume of patients has already been transferred from the public to the private sector. These changes have greatly affected the daily life of public sector pharmacists (for those directly affected), as many public pharmacies have terminated their operations, as mentioned above. The affected public pharmacists were forced to work in Nicosia with different duties and responsibilities (under the management of PhS, HIO or MoH, Nicosia, Cyprus). In addition, the increase in the volume of work in private pharmacies has brought significant changes concerning the functioning of private pharmacies, and generally in the daily life of pharmacists in the private sector.

In this study, an effort was made to explore the determinants of job satisfaction and stress in the context of all these changes. Moreover, the next step is to design those interventions and implement the correct health policies for the management of these phenomena (occupational dissatisfaction and work stress). Only then can a Healthcare System be established that really cares about health professionals with a positive impact on both patients and the state in general. 

## 3. Materials and Methods

A cross-sectional study was conducted involving 419 pharmacists. Among them, 268 pharmacists work in private pharmacies either as owners or as employees, and 151 of the participants are employed in the wider public sector (PhS, SHSO, HIO, MoH-headquarters, Purchasing and Supplying Directorate, and Office of Commissioner of General Healthcare).

The questionnaire was sent electronically to 150 pharmacists (employees in private pharmacies and private pharmacy owners) of the private sector. Among them, 90 responded (60% participation rate). The questionnaire was also distributed to 250 pharmacists throughout Cyprus, of whom 178 responded (71.2% participation rate).

The questionnaire was also distributed to all pharmacists in the public sector (185 in total). For SHSO pharmacists, the questionnaires were given after the head pharmacist in each district was informed. The same procedure was followed for pharmacists working in other sectors. Pharmacists in the public sector responded to a very satisfactory level, with a participation rate of 81.6% (*n* = 185).

The distribution and collection of the questionnaires took place between January and March 2020. Regarding the private sector, convenience sampling was carried out. In the public sector, the questionnaires were distributed to all pharmacists (total population studied).

The studied outcomes were the satisfaction and stress experienced by pharmacists due to their profession. Furthermore, possible determinants of both job satisfaction and stress were investigated. The studied determinants derived from systematic literature review and included the following characteristics: gender, age, marital status, number of children, educational level, years of experience/service, workplace, workload, and interpersonal relationships.

### 3.1. Measuring Tools

The questionnaire contained 2 parts. The first part was related to the recording of social and demographic characteristics of the sample and the second part consisted of structured and weighted questionnaires regarding job satisfaction (the JSS tool) and stress (the PSS tool).

Τhe assessment of job satisfaction was achieved through the use of a structured and weighted questionnaire Job Satisfaction Survey (JSS), as developed by Spector (1985) [31]. Τhe Greek version of the questionnaire was translated and weighted by Tsounis and Sarafis (2018) [32]. For the assessment of stress in pharmacists, the Greek version of the questionnaire the Perceived Stress Scale-14 (PSS-14) was used, which was translated and weighted in Greek by Darviri et al. (2011) [33]. For the selection of the above tools/questionnaires, their validity and reliability were taken into consideration [34].

The questionnaire was accompanied by a cover letter, which referred to information related to the purpose of this study, the confidentiality and anonymity of the data and the voluntary nature of the participation. There was also a reference to the approvals received to conduct this research (positive opinion received by the Cyprus National Bioethics Committee (CNBC), approvals received by the Director of PhS, the Acting Director General of the HIO, the Acting Director of the SHSO, the Scientific Committee for the Promotion of Research-MoH, and the Scientific Council of the SHSO). Completing this questionnaire by the pharmacists was considered as consent to participate in this study.

#### 3.1.1. Tool to Investigate Job Satisfaction

The JSS item scale consists of 36 questions for the evaluation of nine dimensions of job satisfaction. These dimensions are: “pay”, “promotion”, “supervision”, “fringe benefits”, “contingent rewards”, “operating procedures”, “coworkers”, “nature of work”, and “communication” (from 4 declarations each). Pharmacists were requested to respond on a Likert scale as follows: Disagree very much = 1, Disagree moderately = 2, Disagree slightly = 3, Agree slightly = 4, Agree moderately = 5, and Agree very much = 6.

Negatively worded items 2, 4, 6, 8, 10, 12, 14, 16, 18, 19, 21, 23, 24, 26, 29, 31, 32, 34, and 36 were reverse coded (1 = 6, 2 = 5,…, 6 = 1) and, as a result, a higher total score satisfaction (range: 36–216) indicated increased job satisfaction. The internal consistency index Cronbach’s alpha was 0.93 for the JSS scale, a strong indication of the reliability of the scale. Face validity was excellent, as respondents indicated only minor comments.

Some questions from the JSS tool were removed for private pharmacy owners due to the characteristics and nature of their work (not employees). As a result, pharmacy owners were called to answer to the 13 out of 36 questions. Questions 2, 3, 4, 6, 9, 10, 11, 12, 13, 16, 18, 19, 20, 21, 22, 23, 25, 26, 28, 29, 30, 33 and 36 were removed. No other changes were made to the other participants (private pharmacy employees and public sector employees).

#### 3.1.2. Tool to Investigate Perceived Stress 

The PSS item scale includes 14 questions, to which pharmacists were called to answer based on a five-point Likert scale as follows: Never = 0, Almost never = 1, Sometimes = 2, Fairly often = 3, and Very often = 4. Two dimensions are evaluated, self-efficacy and perceived helplessness (7 declarations each).

The total score (range: 0–56) was taken reversing the scores on 7 “positive” questions 4, 5, 6, 7, 9, 10, and 13, i.e., 0 = 4, 1 = 3, 2 = 2, 3 = 1, and 4 = 0, and then adding the scores of all 14 questions. A higher score indicates higher perceived stress. The internal consistency index Cronbach’s alpha was 0.89 for this PSS tool, which indicated the excellent reliability of this tool. The face validity of the tool was exceptional, since no questions, concerns, comments or and clarifications were made by the participants.

### 3.2. Statistical Analysis

Categorical variables—for instance, gender and family status—are presented as frequency (N) and distributions (%), while continuous variables (e.g., age and job satisfaction) are presented as the mean and standard deviation (SD). Internal consistency was evaluated using the Cronbach’s alpha index. Associations between the dimensions of job satisfaction and perceived stress were explored via the Pearson linear correlation coefficient.

Associations between the three groups of respondents (private pharmacy employees, public sector employees, and pharmacy owners), the level of job satisfaction and the level of perceived stress were conducted via analysis of variance (ANOVA). In addition, in the case of two group comparisons, the independent-samples t-test was utilized. The association between the demographic variables and the level of job satisfaction dimensions and the level of the perceived stress dimensions was explored via the multivariate analysis of variance (MANOVA) due to the high correlation between the dimensions [35].

A separated model was fitted for each group of dependent variables—(1) job satisfaction dimensions, and (2) perceived stress dimensions. In the event of the statistically significant association between a demographic variable and the dimension variables, a follow-up univariate ANOVA was performed. When exploring job satisfaction among pharmacy owners, a linear regression model was fitted with total job satisfaction as the dependent variable and the demographic variables as the independent variables, because no dimensions could be extracted since this group only responded to 13 out of 36 items that were relevant to them.

Of 419 participants, 9 (2.15%) participants had missing values in at least one item of the JSS and the PSS. Of 16,442 observations of the JSS and PSS tools, there were 13 (0.08%) missing values. The missing values were imputed with the median value of the variable.

The statistical analysis was conducted using SPSS version 25 and in R version 3.6.1. A *p*-value of less than 0.05 (typically ≤ 0.05) was considered statistically significant.

## 4. Results

### 4.1. Demographic Characteristics

As mentioned above, a cross-sectional study was conducted, involving 419 pharmacists. In the private sector, the participation rate among pharmacists was 67% (*n* = 400, 268/400), and in the wider public sector the participation rate was 81.6% (*n* = 185, 151/185). The overall participation rate in this survey was 71.6% (*n* = 585, 419/585). On average, the completion time for the questionnaire was approximately 5 min, during the participant’s free time.

Table 1 shows the demographic characteristics of the participants. A total of 419 pharmacists participated in this study—72 (17.2%) working as private pharmacy employees, 196 (46.8%) working as owners in private pharmacies, and 151 (36.0%) as working pharmacists in the wider public sector. Among them, 139 participants (33%) were male and 280 (67%) were female, with an average age of 41 years (SD = 12).

### 4.2. Internal Consistency of Tools

The internal consistency of the tools was high. The internal consistency index Cronbach’s alpha for the JSS tool was 0.93, and 0.89 for the PSS tool. 

### 4.3. Descriptive Statistics

Table 2 shows the mean score (standard deviation) in each dimension of job satisfaction and perceived stress for all the study participants.

**Job satisfaction**

Regarding the nine dimensions of job satisfaction, it was generally observed that the employees in private pharmacies had the highest average level of job satisfaction in all dimensions. A statistically significant difference between these two categories was observed in all dimensions (*p* < 0.05), except for the supervision dimension (*p* = 0.3).

**Perceived stress**

A statistically significant difference was observed among three categories regarding the mean level of self-efficacy (*p* < 0.001)—while 419 participants were at 17.5 (4.7), private pharmacy employees had an average of 15.7 (4.7), pharmacy owners had an average of 17.5 (4.3) and public sector employees had an average of 18.2 (4.9). Pharmacists in the wider public sector had the highest mean score of self-efficacy compared to the other categories.

### 4.4. Pharmacy Owners

Job satisfaction had a high and negative correlation with perceived stress (r = −0.51, *p* < 0.01), a high and positive correlation with self-efficacy (r = 0.43, *p* < 0.01), and a high and negative correlation with perceived helplessness (r = −0.48, *p* < 0.01). Perceived stress dimensions had a high correlation among each other.

The results of the MANOVA show that gender is statistically significantly associated (Wilks’ lambda = 0.946, *p* = 0.006) with the PSS scale for perceived stress.

The follow-up univariate ANOVA for the impact of gender showed that gender is associated with the dimension of perceived helplessness (*p* = 0.001). Female pharmacists, with a mean perceived helplessness score of 17.8 (SD = 5.3), have a higher score compared to male pharmacists, with a mean helplessness score of 14.3 (SD = 5.1).

### 4.5. Pharmacists Working in the Wider Public Sector

The pharmacists working under the management of the Ministry of Health (MoH, Purchasing and Supplying Directorate) had lower levels of job satisfaction in comparison to pharmacists working in other organizations (HIO, SHSO, and PhS). This applies in all dimensions.

In addition to this, pharmacists under the management of the SHSO (hospitals and health centers) had the least total perceived stress compared to pharmacists in other workplaces. They had the highest self-efficacy and the lowest perceived helplessness (Table 3).

The hospital pharmacists and the pharmacists who work in the health centers under the management of the SHSO had higher job satisfaction regarding supervision (*p* = 0.011), the operating procedures (*p* = 0.006) and the nature of work (*p* = 0.006). They also had lower perceived stress (*p* = 0.008) in total. The pharmacists who work under the management of the SHSO showed higher self-efficacy (*p* = 0.006) and lower perceived helplessness (*p* = 0.031) (Table 4).

The pharmacists working under the management of the MoH had lower job satisfaction in total (=95) in every dimension of job satisfaction and showed the highest perceived stress (statistically not significant, *p* = 0.7) (Table 5).

Job satisfaction had a high negative correlation with perceived stress (r = −0.45, *p* < 0.01), a moderate positive correlation with self-efficacy (r = 0.30, *p* < 0.01) and a high negative correlation with perceived helplessness (r = −0.51, *p* < 0.01). Overall perceived stress had moderate negative correlation with all subdimensions of job satisfaction.

Table 6 shows the results of a MANOVA on the impact of demographic characteristics on the dimensions of the JSS of pharmacists/employees in the wider public sector. The results showed that age had an impact on the dimensions of job satisfaction (Wilks’ lambda = 0.859, *p* = 0.011), as well as the workplace (HIO, SHSO, MoH, and PhS) (Wilks’ lambda = 0.421, *p* < 0.001).

The univariate ANOVA for the impact of age in every dimension of job satisfaction of employees in the public sector showed that age had an impact on the level of job satisfaction regarding the promotion (*p* = 0.031). It appeared that there is a weak positive correlation of age and satisfaction with promotion (r = 0.18, *p* = 0.031), i.e., increased age is slightly associated with increased satisfaction from the dimension of the promotion.

Table 7 shows the univariate ANOVA for the impact of the workplace in every dimension of job satisfaction for public sector employees. The univariate analysis in the Table 7 showed that the workplace had an effect on almost every dimension of job satisfaction, except the dimension of “pay”.

Table 8 presents the results of a MANOVA on the impact of the demographic characteristics of public sector employees in relation to the dimensions of the PSS. It appeared that gender (Wilks’ lambda = 0.899, *p* = 0.001), age (Wilks’ lambda = 0.933, *p* = 0.010) and whether the employee had children (Wilks’ lambda = 0.944, *p* = 0.021) affected the dimensions of perceived stress.

Table 9 shows the results of a univariate ANOVA on the impact of gender, age, and marital status (having children) on the level of dimensions of perceived helplessness and self-efficacy.

**Age**

Age had an impact on both self-efficacy (*p* = 0.005) and perceived helplessness (*p* = 0.006) (Table 9).

Further analysis showed that there is a low positive correlation of age and self-efficacy (*r* = 0.23, *p* = 0.0057), and a low negative correlation with perceived helplessness (*r* = −0.21, *p* = 0.009). That is, older age was associated with greater self-efficacy and less perceived helplessness.

**Marital status**

Whether an employee has children had an impact not only on self-efficacy (*p* = 0.039) but on perceived helplessness as well (*p* = 0.006) (Table 9).

Further analysis on the average level of self-efficacy and perceived helplessness between people who have children and those who do not indicated that:

- People with children had a greater level of self-efficacy [19.1 (SD = 4.6)] compared to people with no children [16.5 (SD = 5.1)].
- People who have children had lower levels of perceived helplessness [14.6 (SD = 5.2)] compared to people with no children [18.0 (SD = 5.6)].

**Gender**

Gender only affected perceived helplessness (p=0.001) (Table 9). Further analysis of the average level of perceived helplessness showed that female pharmacists had an average level of 16.6 (SD = 5.5), far greater than male pharmacists who had an average level of 13 (SD = 4.9).

### 4.6. Employees in Private Pharmacies

Table 10 compares pharmacists who work under the management of the SHSO and pharmacists who are employees in private pharmacies.

Employees in private pharmacies appear to have a greater total job satisfaction (*p* < 0.001). In terms of the dimensions of job satisfaction, private pharmacy employees have a greater satisfaction regarding pay (*p* = 0.005), promotion (*p* < 0.001), fringe benefits (*p* = 0.007), coworkers (*p* < 0.007), and communication (*p* < 0.001).

Employees who work in the private sector, however, have increased perceived stress in comparison to pharmacists who work under the management of the SHSO (*p* < 0.001), as well as lower self-efficacy (*p* < 0.001).

Job satisfaction had a moderate negative association with perceived stress (r = −0.29, *p* < 0.01), a moderate positive association with self-efficacy (r = 0.40, *p* < 0.01) and a low negative association with perceived helplessness (r = −0.15, *p* < 0.01). Total perceived stress showed lower to moderate negative correlations with all subdimensions of job satisfaction.

## 5. Discussion

In this study, an effort was made to investigate the factors associated with job satisfaction and stress in pharmacists both in the public and the private sectors in Cyprus. This is the second study conducted to address this specific issue. This is, however, the first research study covers pharmacists working in private pharmacies and also those working in the wider public sector.

The previous study carried out in 2013 by Papanicolaou G. [36], concerned pharmacists working in public hospitals in Cyprus, with a coverage rate of 61.5%. In the present research, the Pancyprian coverage of the sample (400 questionnaires were distributed in the private and 185 in the public sector, total 585) combined with the high participation rate of pharmacists (67% in the private sector, 81.6% in the public sector, in total 71.6%) ensure both the representativity of the sample as well as the possibility of generalizing the results.

Regarding private pharmacy owners and considering the results of the MANOVA, it appeared that from the demographic characteristics, only gender was statistically significantly associated with the PSS tool (investigation of perceived stress). After the univariate ANOVA, gender appeared only to affect perceived helplessness (*p* = 0.001).

Female pharmacists/pharmacy owners (mean score: 17.8) had a higher level of perceived helplessness compared to male pharmacists (mean score: 14.3). Regarding the pharmacists who work in the wider public sector, it appeared that gender also affected the dimensions of perceived stress (MANOVA). According to the results of the univariate ANOVA, gender was found to only have an effect on perceived helplessness (*p* = 0.001). Further analysis on the mean score of perceived helplessness showed that female pharmacists had a mean score of 16.6, far greater than male pharmacists who had 13. As far as the employees in private pharmacies are concerned, it appeared that the demographic characteristics had no impact on the dimensions of job satisfaction. This is also confirmed by other surveys.

In a study by McCann and associates (2009) [11], it was found that there was a statistically significant difference regarding stress between female and male pharmacists, with female pharmacists appearing more vulnerable to stress (21.70 vs. 20.40, *p* = 0.001). Yeh Y.-C. and associates (2009) concluded that female pharmacists suffered with insomnia, caused by demanding professions and a high level of stress more often than male pharmacists [37]. The finding of the Carvajal et al. (2019) study in the USA is in full agreement with the finding of the present study, namely the fact that female pharmacists experienced more stress than male pharmacists (*p* ≤ 0.001) [38].

Taking into consideration other research papers conducted in other areas with various health professionals, it is assumed that female employees perceive more stress due to the absence of adequate stress management mechanisms and because of difficulty in finding a balance between family and work life [39,40]. As a result, male pharmacists are bound to experience a severe conflict of roles [39,40,41]. Three studies found that female pharmacists overall expressed higher levels of job satisfaction in comparison to male pharmacists [26,38,42]. Regarding the JSS tool used in the present study for job satisfaction, no significant statistical conclusion was reported.

This study has observed that, in general, pharmacists working under the management of the MoH had lower levels of job satisfaction than the pharmacists of other organizations (HIO, SHSO, and PhS). This was apparent in every dimension (pay, promotion, supervision, fringe benefits, contingent rewards, operating procedures, coworkers, nature of work and communication). Furthermore, the pharmacists working under the management of the SHSO (hospitals and health centers) had less overall perceived stress compared to pharmacists in other workplaces. They had higher self-efficacy and lower perceived helplessness. The pharmacists working in hospitals and health centers under the management of the SHSO had higher job satisfaction regarding supervision (*p* = 0.011), operating procedures (*p* = 0.006) and nature of work (*p* = 0.006) than pharmacists working in other organizations (HIO, MoH, and PhS).

When a comparison was made between office workplaces (HIO, MoH, and PhS), it appeared that pharmacists working under the management of the MoH had the lowest total job satisfaction in all dimensions of job satisfaction and showed higher perceived stress. Concerning the comparison of private pharmacy employees and employees working in the SHSO (public pharmacies), it appeared that private pharmacy employees had higher levels of job satisfaction (*p* < 0.001), while at the same time they had higher levels of perceived stress regarding the pharmacists working in public hospitals (*p* < 0.001). It is presumed that the workplace plays a significant role in terms of job satisfaction and stress. This was also evident through literature. Several articles showed the significance of the workplace (community pharmacists, hospital pharmacists, etc.) in terms of job satisfaction and stress. In a study by Lin and associates (2007) in Taiwan, it appeared that community pharmacists were more satisfied with their job than hospital pharmacists [43]. Additionally, in another extensive study by McCann and associates (2009) which was conducted in North Ireland, the total score in the stress assessment scale was greater in community pharmacists compared to hospital pharmacists (*p* < 0.05) [11]. The above studies are in line with the findings of the present study. In contrast, in three other surveys, the results were different, with community pharmacists appearing less satisfied [26,44,45]. The studies involved 32,181, 303 and 235 pharmacists in Great Britain, the USA, and Jordan, respectively. Another study by Ortmeier and Wolfgang (1991) in the USA with 327 participants, showed that workplace as well as responsibilities played a significant role regarding job satisfaction in pharmacists (*p* < 0.02) [19]. It was considered that community pharmacists had increased stress (in line with the findings of the present study) and low levels of job satisfaction (in contrast with the findings of the present study) due to the career plan created by each pharmacist and low commitment to work.

In regard to the public sector pharmacists, it appeared that age had an impact on job satisfaction. A weak positive association between age and satisfaction due to promotion (*p* = 0.031) was found. The present study also showed that age had an impact on both self-efficacy (*p* = 0.005) and perceived helplessness (*p* = 0.006). Older age was associated with greater self-efficacy and less perceived helplessness. This can also be seen in the literature. Several studies showed the correlation of age with respect to the outcomes of the present study (job satisfaction, work stress). Additionally, several studies appear to agree on the existence of a positive correlation of age with the work outcomes of pharmacists. A study by Ortmeier and Wolfgang (1991) reports that as age increases, the perception of job satisfaction appears to improve (*p* < 0.002) [19]. In parallel, another study by Yeh and associates (2009) in Taiwan, involving 247 hospital pharmacists, showed that age and years in the profession had a positive impact in terms of work stress (r = 0.82, r = 0.75, *p* < 0.001) [35]. In addition, another study by Teong et al. (2019), involving 286 community pharmacists (*p* < 0.001), came to the same conclusion [46]. Three other studies carried out in 2011 (Liu and White), 2015 (Manan et al.) and 2018 (Gustafsson et al.) concluded that job satisfaction increased with increasing age [47,48,49].

In the present study, it was found that having children (marital status) had an impact on both self-efficacy (*p* = 0.039) and perceived helplessness (*p* = 0.006). In more detail, pharmacists with children showed a greater level of self-efficacy and a lower level of perceived helplessness in comparison to pharmacists without children. Therefore, the total level of perceived stress is also reduced. A study by Mott et al. (2004) showed that the marital status affected work attitudes and behaviors [50]. Pharmacists who had children aged 13–17 (15.3) had higher levels of work stress than their colleagues who did not have children (14.6), apparently because of their many responsibilities, concerns about their household, and because they also had to deal with the sensitive adolescent years of their children (*p* < 0.05). Pharmacists with children aged > 17 appeared to have higher levels of job satisfaction (*p* < 0.05) and lower levels of work stress (*p* < 0.05) (in line with the finding of this study). In another study by Gidman et al. (2007), which took place in the United Kingdom and involved 30 female community pharmacists (qualitative research), those who had children reported higher levels of work stress and that they had a greater role in childcare than their partners. Τhe aforementioned study found some evidence that increased workload, as female community pharmacists, resulted in reduced well-being and health [51]. Thus, having children appears to affect pharmacists’ ability to find a balance in their daily lives.

In this study, regarding private pharmacy owners, it was shown that job satisfaction had a high negative correlation with perceived stress (*p* < 0.01), a high positive correlation with self-efficacy (*p* < 0.01) and a high negative correlation with perceived helplessness (*p* < 0.01). This can also be observed in the public sector pharmacists, with the only difference being that there is a moderate positive correlation of job satisfaction with self-efficacy. In private pharmacy employees, job satisfaction was found to have a moderate negative correlation with perceived stress (*p* < 0.01), a moderate positive correlation with self-efficacy (*p* < 0.01) and a low negative correlation with perceived helplessness (*p* < 0.01). This negative correlation of job satisfaction and stress can also be seen in the literature. In a study by McCann et al. in Northern Ireland which involved 766 pharmacists, it was concluded that pharmacists’ job satisfaction constitutes a positive factor in the management of work stress [11]. In addition, a study by Gaither et al. (2008) in the USA including 2250 pharmacists found a negative correlation of work stress and job satisfaction (−0.12) [12].

Summing up, the total job satisfaction score of the employees in a private pharmacy was 144, while it was 122 (minimum: 36; maximum: 216) among the public sector employees. Pharmacy owners in the private sector, in the total of 13 declarations, had an average level of job satisfaction of 47 (minimum: 13; maximum: 78). In terms of the nine dimensions of job satisfaction, the employees in private pharmacies had a higher average level of job satisfaction in all dimensions. On the other hand, the average total perceived stress in a total of 419 participants was 27 (minimum: 0; maximum: 56). No statistically significant difference in the total perceived stress between the three categories was observed. Regarding the mean level of self-efficacy, a statistically significant difference was observed (*p* < 0.001), with the employees in the wider public sector having a higher mean score (=18.2) compared to private pharmacy owners (=17.5) and the private pharmacy employees (=15.7).

## 6. Limitations

This study was conducted in a specific time frame (January–March 2020) which happened to co-exist with the implementation of the GHS (Phase A). Consequently, there is no recorded knowledge and experience of the situation prior to the implementation of the GHS, especially regarding private pharmacies. For instance, employees in private pharmacies were observed to have a higher mean level of job satisfaction in all dimensions compared to the employees at the wider public sector. However, it was found that they had the highest level of total perceived stress (=28), compared to pharmacy owners (=27) and pharmacists in the wider public sector (=26). No statistically significant difference was observed. Where a statistically significant difference was observed, it was with reference to one dimension of the PSS, the mean level of self-efficacy (*p* < 0.001). The lowest mean level of self-efficacy was shown in private pharmacy employees (=15.7) compared to private pharmacy owners (=17.5) and pharmacists working in the wider public sector (=18.2).

Another limitation of this study is the fact that the JSS questionnaire given to private pharmacy owners was shorter than the rest of the studied population. Because of the characteristics and the nature of their job (not employees), some questions had to be removed from this tool. Pharmacists who owned their own pharmacy belong to the category of self-employed entrepreneurs. Consequently, this affected the comparison with the other categories, since the minimum and maximum possible job satisfaction of pharmacy owners was 13 and 78, respectively. In contrast, the minimum and maximum possible total job satisfaction for the other categories of employees were 36 and 216, respectively.

In this study, it is not considered unlikely that some questions in the questionnaire were not fully perceived, although an attempt was made to avoid such errors (the main researcher’s phone number was given for needed clarification in the participant information sheet/cover letter). Moreover, since both job satisfaction and perceived stress were affected not only by labor but also by personal factors, it is likely that the latter have had a certain degree of impact regarding the participants’ responses.

## 7. Conclusions

In this research, it was shown that job satisfaction in pharmacists had a negative correlation with perceived stress, a positive correlation with self-efficacy and a negative correlation with perceived helplessness. At the same time, it became apparent that the stress levels among pharmacists working in private pharmacies (either as employees or as owners) are higher compared to pharmacists working in the wider public sector. This is likely to be due to the increasing workload, the long working hours, the increase in public turnout, the need to recruit additional staff, etc., brought by the implementation of the GHS. Support for pharmacists is considered necessary, through programs, etc., for the proper management of the work stress that they experience.

## 8. Proposals

The constant developments in the health sector require a change in direction by the administrations running pharmacies and other organizations and associations, focusing on aspects of the profession that make workers happy, satisfied and less stressed [12]. Proper workload training, stress management, time management, implementation of pharmaceutical care and communication (fostering empathy) can increase both job satisfaction and the efficiency of workers [45]. In the long term, the results regarding the administration of pharmacies will be beneficial in terms of profit and competitiveness.

In parallel, employees should be motivated through, for example, rewards and promotions. Evaluation criteria need to be constantly updated in order to ensure a sense of fairness and fair competition. The operating procedures need review and flexibility to some extent, in order to increase not only productivity but employee efficiency as well. It is undeniably essential to develop support programs for pharmacists emphasizing the introduction of mechanisms for managing work stress [52].

In conclusion, limited knowledge of the changes leads to uncertainty regarding the future of the pharmacy profession with levels of stress among pharmacists increasing [11]. The participation of pharmacists in the decision-making centers that affect the health sector is considered crucial for the achievement of optimal results and to provide quality services in the best interest of the citizen [53].

## Figures and Tables

**Table 1:** Demographic characteristics of participants (N = 419).

	Total Number N = 419	Employee in a Private Pharmacy N = 72	Private Pharmacy Owner N = 196	Public Sector Employee N = 151
**1. Gender**				
Male	139 (33%)	16 (22%)	88 (45%)	35 (23%)
Female	280 (67%)	56 (78%)	108 (55%)	116 (77%)
**2. Age**	Mean: 41 (SD: 12)	Mean: 30 (SD: 8)	Mean: 44 (SD: 12)	Mean: 42 (SD: 10)
(unknown)	3	0	0	3
**3. Marital status**				
Single	113 (27%)	41 (57%)	41 (21%)	31 (21%)
Married	247 (59%)	12 (17%)	132 (67%)	103 (69%)
Symbiosis	42 (10%)	15 (21%)	17 (8.7%)	10 (6.7%)
Widowed	4 (1%)	0 (0%)	2 (1%)	2 (1.3%)
Divorced	12 (2.9%)	4 (5.6%)	4 (2%)	4 (2.7%)
(unknown)	1	0	0	1
**4. Having children**				
Yes	246 (59%)	12 (17%)	131 (67%)	103 (69%)
No	172 (41%)	60 (83%)	65 (33%)	47 (31%)
(unknown)	1	0	0	1
**5. Number of children**	Mean: 2.07 (SD: 0.91)	Mean: 1.67 (SD: 0.49)	Mean: 2.12 (SD: 0.89)	Mean: 2.05 (SD: 0.97)
	n = 173	n = 60	n = 65	n = 48
**6. Year of starting the profession**	Mean: 2004 (SD: 12)	Mean: 2015 (SD: 7)	Mean: 2001 (SD: 12)	Mean: 2002 (SD: 10)
(unknown)	3	0	0	3
**7. Education level**				
Master degree	163 (39%)	16 (22%)	62 (32%)	85 (57%)
PhD	6 (1.4%)	0 (0%)	3 (1.5%)	3 (2%)
Master degree/PhD	163 (39%)	16 (22%)	62 (32%)	85 (56%)
**8. Work district**				
Famagusta	23 (5.5%)	3 (4.2%)	16 (8.2%)	4 (2.6%)
Larnaca	44 (11%)	14 (19%)	24 (12%)	6 (4.0%)
Limassol	97 (23%)	22 (31%)	63 (32%)	12 (7.9%)
Nicosia	212 (51%)	30 (42%)	62 (32%)	120 (79%)
Paphos	43 (10%)	3 (4.2%)	31 (16%)	9 (6.0%)
**9. Work area**				
Rural	49 (12%)	12 (17%)	29 (15%)	8 (5.3%)
Urban	370 (88%)	60 (83%)	167 (85%)	143 (95%)
**10. Workplace**				
HIO				12 (8%)
SHSO				54 (36%)
MoH				18 (12%)
PhS				67 (44%)
**11. Duration of the occupation (years)**	Mean: 16 (SD: 12)	Mean: 5 (SD: 7)	Mean: 19 (SD: 12)	Mean: 18 (SD: 10)
(unknown)	3	0	0	3

**Table 2:** Mean score (standard deviation) of tools and dimensions.

Dimension	Ν	Total	Employee in a Private Pharmacy N = 72	Private Pharmacy Owner N = 196	Public Sector Employee N = 151	*p*-Value
*Total job satisfaction*	419	91 (47)	144 (30)	47 (10) *	122 (30)	**<0.001 ^†^**
Pay	223	12.7 (5.1)	14.4 (5.0)		11.9 (5.0)	**<0.001**
Promotion	223	9.1 (4.6)	11.3 (4.8)		8.1 (4.2)	**<0.001**
Supervision	223	19.6 (5.2)	20.2 (4.6)		19.4 (5.5)	0.3
Fringe benefits	223	12.0 (5.0)	13.3 (4.9)		11.3 (5.0)	**0.006**
Contingent rewards	223	13.1 (5.5)	16.3 (5.5)		11.6 (4.8)	**<0.001**
Operating procedures	223	11.4 (4.3)	12.4 (4.2)		11.0 (4.2)	**0.024**
Coworkers	223	19.1 (4.1)	19.9 (3.7)		18.7 (4.2)	**0.037**
Nature of work	223	17.4 (4.4)	18.5 (3.9)		16.9 (4.5)	**0.013**
Communication	223	14.3 (5.6)	17.6 (4.7)		12.7 (5.4)	**<0.001**
*Total perceived stress*	419	27 (9)	28 (9)	27 (9)	26 (10)	0.071
Self-efficacy	419	17.5 (4.7)	15.7 (4.7)	17.5 (4.3)	18.2 (4.9)	**<0.001**
Perceived helplessness	419	16.2 (5.5)	16.1 (5.8)	16.7 (5.4)	15.7 (5.6)	0.3

* Calculated in 13/36 tool declarations. Not directly comparable in two groups: private and public sector employees. ^†^ *t*-test between private and public sector employees. *T-test for total job satisfaction and dimensions; ANOVA test for total perceived stress and dimensions.*

**Table 3:** Mean score (standard deviation) in the dimensions of job satisfaction and perceived stress among four workplaces regarding the pharmacists in the wider public sector.

	HIO, N = 12	SHSO (Hospitals and Health Centers) N = 54	Ministry of Health N = 18	Pharmaceutical Services N = 67	*p*-Value *
*Total job satisfaction*	139 (21)	122 (23)	95 (24)	125 (33)	**<0.001**
Pay	11.8 (4.7)	11.9 (4.8)	10.3 (4.0)	12.4 (5.5)	0.5
Promotion	8.9 (3.9)	7.3 (3.2)	6.1 (2.6)	9.1 (4.9)	**0.015**
Supervision	22.0 (3.0)	20.9 (4.3)	12.8 (6.8)	19.4 (5.2)	**<0.001**
Fringe benefits	15.3 (6.1)	10.9 (4.8)	10.1 (4.0)	11.3 (4.9)	**0.024**
Contingent rewards	14.7 (4.7)	10.9 (4.0)	8.4 (4.1)	12.4 (5.2)	**0.001**
Operating procedures	10.9 (2.9)	12.2 (4.0)	7.1 (3.0)	11.0 (4.3)	**<0.001**
Coworkers	21.5 (2.9)	18.0 (4.0)	16.1 (5.0)	19.4 (3.8)	**<0.001**
Nature of work	18.7 (3.5)	18.3 (4.1)	14.5 (5.1)	16.2 (4.4)	**0.003**
Communication	14.8 (4.8)	11.6 (5.2)	9.6 (5.2)	14.1 (5.2)	**0.002**
*Total perceived stress*	28 (8)	23 (9)	29 (10)	26 (10)	**0.048**
Self-efficacy	16.2 (4.2)	19.7 (4.5)	17.3 (5.1)	17.7 (5.1)	**0.037**
Perceived helplessness	16.2 (4.6)	14.4 (5.5)	18.0 (5.9)	16.1 (5.5)	0.10

* ANOVA test.

**Table 4:** Mean score (standard deviation) in the dimensions of job satisfaction and perceived stress between the SHSO and the other three office workplaces (HIO, MoH, and PhS).

	SHSO N = 54	Other Organizations (HIO, MoH, and PhS) N = 97	*p*-Value *
*Total job satisfaction*	122 (23)	121 (33)	>0.9
Pay	11.9 (4.8)	11.9 (5.2)	>0.9
Promotion	7.3 (3.2)	8.5 (4.6)	0.082
Supervision	20.9 (4.3)	18.5 (6.0)	**0.011**
Fringe benefits	10.9 (4.8)	11.6 (5.1)	0.4
Contingent rewards	10.9 (4.0)	11.9 (5.2)	0.2
Operating procedures	12.2 (4.0)	10.3 (4.2)	**0.006**
Coworkers	18.0 (4.0)	19.1 (4.2)	0.14
Nature of work	18.3 (4.1)	16.2 (4.5)	**0.006**
Communication	11.6 (5.2)	13.4 (5.4)	0.051
*Total perceived stress*	23 (9)	27 (10)	**0.008**
Self-efficacy	19.7 (4.5)	17.4 (5.0)	**0.006**
Perceived helplessness	14.4 (5.5)	16.5 (5.5)	**0.031**

* *t*-test.

**Table 5:** Mean score (standard deviation) in the dimensions of job satisfaction and total perceived stress among the three office workplaces (HIO, MoH, and PhS).

	HIO N = 12	MoH N = 18	Ph. Services N = 67	*p*-Value *
*Total job satisfaction*	139 (21)	95 (24)	125 (33)	**<0.001**
Pay	11.8 (4.7)	10.3 (4.0)	12.4 (5.5)	0.3
Promotion	8.9 (3.9)	6.1 (2.6)	9.1 (4.9)	**0.049**
Supervision	22.0 (3.0)	12.8 (6.8)	19.4 (5.2)	**<0.001**
Fringe benefits	15.3 (6.1)	10.1 (4.0)	11.3 (4.9)	0.015
Contingent rewards	14.7 (4.7)	8.4 (4.1)	12.4 (5.2)	**0.002**
Operating procedures	10.9 (2.9)	7.1 (3.0)	11.0 (4.3)	**0.001**
Coworkers	21.5 (2.9)	16.1 (5.0)	19.4 (3.8)	**<0.001**
Nature of work	18.7 (3.5)	14.5 (5.1)	16.2 (4.4)	**0.045**
Communication	14.8 (4.8)	9.6 (5.2)	14.1 (5.2)	**0.004**
*Total perceived stress*	28 (8)	29 (10)	26 (10)	0.7
Self-efficacy	16.2 (4.2)	17.3 (5.1)	17.7 (5.1)	0.6
Perceived helplessness	16.2 (4.6)	18.0 (5.9)	16.1 (5.5)	0.4

* ANOVA test.

**Table 6:** MANOVA for the impact of demographic characteristics on the dimensions of the JSS of the employees working in the wider public sector.

Factor	Wilks’ Lambda	*f*-Value	*p*-Value
Gender	0.940	0.901	0.527
Age	0.849	2.518	**0.011**
With children	0.910	1.401	0.195
Years of experience	0.924	1.154	0.330
Marital status	0.821	0.962	0.522
Education	0.879	1.938	0.052
Area of residence	0.900	1.571	0.131
Workplace	0.421	4.750	**<0.001**

**Table 7:** Univariate ANOVA for the impact of the workplace in all dimensions of job satisfaction of employees working in the wider public sector.

Dimension	(Sum. Square)	(Mean Sq. Error)	*f*-Value	*p*-Value
Pay	89.157	29.719	1.183	0.319
Promotion	302.794	100.931	7.134	**<0.001**
Supervision	888.445	296.148	12.468	**<0.001**
Fringe benefits	292.908	97.636	4.226	**0.007**
Contingent rewards	427.838	142.613	7.004	**<0.001**
Operating procedures	303.160	101.053	6.786	**<0.001**
Coworkers	427.661	142.554	9.685	**<0.001**
Nature of work	251.021	83.674	4.504	**0.005**
Communication	476.471	158.824	6.375	**<0.001**

**Table 8:** MANOVA on the impact of the demographic characteristics of public sector employees on the dimensions of the PSS.

Factor	Wilks’ Lambda	*f*-Value	*p*-Value
Gender	**0.899**	7.546	**0.001**
Age	**0.933**	4.802	**0.010**
Having children	**0.944**	3.953	**0.021**
Years of experience	0.984	1.073	0.345
Marital status	0.957	0.992	0.431
Education	0.993	0.447	0.641
Area of residence	0.996	0.287	0.751
Workplace	0.916	2.002	0.066

**Table 9:** Univariate ANOVA on the impact of age, marital status and gender, on the level of dimensions of perceived stress on public sector employees.

	(Sum. Sq.)	(Mean Sq. Error)	*f*-Value	*p*-Value
**Age**				
Self-efficacy	180.915	180.915	8.137	**0.005**
Perceived helplessness	201.395	201.395	7.882	**0.006**
**With children**				
Self-efficacy	96.801	96.801	4.354	**0.039**
Perceived helplessness	200.744	200.744	7.857	**0.006**
**Gender**				
Self-efficacy	31.339	31.339	1.410	0.237
Perceived helplessness	327.760	327.760	12.828	**0.001**

**Table 10:** Mean score (standard deviation) in the dimensions of job satisfaction between pharmacists who work under the management of the SHSO and private pharmacy employees.

	Private Pharmacy Employees N = 72	Pharmacists in the SHSO N = 54	*p*-Value *
*Total job satisfaction*	144 (30)	122 (23)	**<0.001**
Pay	14.4 (5.0)	11.9 (4.8)	**0.005**
Promotion	11.3 (4.8)	7.3 (3.2)	**<0.001**
Supervision	20.2 (4.6)	20.9 (4.3)	0.4
Fringe benefits	13.3 (4.9)	10.9 (4.8)	**0.007**
Contingent rewards	16.3 (5.5)	10.9 (4.0)	**<0.001**
Operating procedures	12.4 (4.2)	12.2 (4.0)	0.9
Coworkers	19.9 (3.7)	18.0 (4.0)	**0.007**
Nature of work	18.5 (3.9)	18.3 (4.1)	0.8
Communication	17.6 (4.7)	11.6 (5.2)	**<0.001**
*Total perceived stress*	28 (9)	23 (9)	**<0.001**
Self-efficacy	15.7 (4.7)	19.7 (4.5)	**<0.001**
Perceived helplessness	16.1 (5.8)	14.4 (5.5)	0.11

* *t*-test.

## Data Availability

The data that support the findings of this study are available from the corresponding author, G.S., upon reasonable request.

## Abbreviations

Job Satisfaction Survey (JSS), Perceived Stress Scale-14 (PSS-14), Ministry of Health (MoH), State Health Services Organization (SHSO), General Healthcare System (GHS), Pharmaceutical Services (PhS), Health Insurance Organization (HIO), Cyprus National Bioethics Committee (CNBC), standard deviation (SD), analysis of variance (ANOVA), and multivariate analysis of variance (MANOVA).

## References

[1] McEwen B. (2005). Stressed or stressed out: What is the difference?. J. Psychiatry Neurosci..

[2] Michie S. (2002). Causes and management of stress at work. Occup. Environ. Med..

[3] Clegg S.R., Hardy C., Nord W.R. (1996). Handbook of Organisation Studies.

[4] Porac J.F., Rowland K.M., Ferris G.R. (1987). The job satisfaction questionnaire as a cognitive event: First- and second- order processes in affective commentary. Research in Personnel and Human Resources Management.

[5] Cranny C.J., Smith P.C., Stone E.F. (1992). Job Satisfaction: How People Feel about their Jobs and How It Affects Their Performance.

[6] Price J.L. (2001). Reflections on the determinants of voluntary turnover. Int. J. Manag..

[7] Locke E.A., Dunnette M.D. (1976). The nature and causes of job satisfaction. Handbook of Industrial and Organizational Psychology.

[8] Rice R.W., Near J.P., Hunt R.G. (1980). The job-satisfaction/life-satisfaction relationship: A review of empirical research. Basic Appl. Soc. Psychol..

[9] Weiss H.M., Cropanzano R., Staw B.M., Cummings L.L. (1996). Affective Events Theory: A Theoretical Discussion of the Structure, Causes and Consequences of Affective Experiences at Work. Research in Organizational Behavior: An Annual Series of Analytical Essays and Critical Reviews.

[10] McCann L., Adair C.G., Hughes C.M. (2009). An exploration of work-related stress in Northern Ireland community pharmacy: A qualitative study. Int. J. Pharm. Pract..

[11] McCann L., Hughes C.M., Adair C.G., Cardwell C. (2009). Assessing job satisfaction and stress among pharmacists in Northern Ireland. Pharm. World Sci..

[12] Gaither C.A., Kahaleh A.A., Doucette W.R., Mott D.A., Pederson C.A., Schommer J.C. (2008). A modified model of pharmacists’ job stress: The role of organizational, extra-role, and individual factors on work-related outcomes. Res. Soc. Adm. Pharm..

[13] Mott D.A. (2000). Pharmacist job turnover, length of service, and reasons for leaving, 1983–1997. Am. J. Health-Syst. Pharm..

[14] Gaither C.A. (1999). Career Commitment: A Mediator of the Effects of Job Stress on Pharmacists’ Work-Related Attitudes. J. Am. Pharm. Assoc..

[15] Wolfgang A.P. (1987). Is pharmacist turnover a function of job stress?. Am. Pharm..

[16] Wolfgang A.P., Kirk K.W., Shepherd M.D. (1985). Job stress in pharmacy practice: Implications for managers. Am. Pharm..

[17] Wolfgang A.P., Perri M., Wolfgang C.F. (1988). Job-related stress experienced by hospital pharmacists and nurses. Am. J. Hosp. Pharm..

[18] Okoye N.V., Obono M.O. (2012). Stress and Coping Strategies among Hospital Pharmacists in Lagos state, Nigeria. West. Afr. J. Pharm..

[19] Ortmeier B.G., Wolfgang A.P. (1991). Job-Related Stress: Perceptions of Employee Pharmacists. Am. Pharmacy..

[20] Stewart J.E., Smith S.N. (1987). Work expectations and organizational attachment of hospital pharmacists. Am. J. Hosp. Pharm..

[21] Firth-Cozens J., Payne R. (1999). Stress in Health Professionals.

[22] McNeely E. (2005). The consequences of job stress for nurses’ health: Time for a check-up. Nurs. Outlook.

[23] Royal Pharmaceutical Society of Great Britain (2006). Stress: It’s not just Patients Who Get Stressed.

[24] Royal Pharmaceutical Society RPS Guidance on Ethical, Professional Decision Making in the COVID-19 Pandemic. https://www.rpharms.com/Portals/0/RPS%20document%20library/Open%20access/Coronavirus/00239%20001a%202004%20COVID19%20Ethical%20guide%20document%20WEB.pdf?ver=2020-04-09-111739-353.

[25] Lapane K.L., Hughes C.M. (2004). Baseline job satisfaction and stress among pharmacists and pharmacy technicians participating in the Fleetwood Phase III study. Consult Pharm..

[26] Seston E., Hassell K., Ferguson J., Hann M. (2009). Exploring the relationship between pharmacists’ job satisfaction, intention to quit the profession, and actual quitting. Res. Soc. Adm. Pharm..

[27] Hibbert D., Bissell P., Ward P. (2002). Consumerism and professional work in the community pharmacy. Sociol. Health Illness.

[28] Boardman H., Blenkinsopp A., Jesson J., Wilson K. (2001). A pharmacy workforce survey in the west midlands: Morale and motivation. Pharm. J..

[29] Theodorou M., Charalambous C., Petrou C., Cylus J. (2012). Cyprus: Health system review. Health Syst. Transit..

[30] GHS: Eligibility—About Pharmacies. https://www.gesy.org.cy/sites/Sites?d=Desktop&locale=en_US&lookuphost=/en-us/&lookuppage=hiopharmacieseligibility.

[31] Spector P.E. (1985). Measurement of human service staff satisfaction: Development of the Job Satisfaction Survey. Am. J. Community Psychol..

[32] Tsounis A., Sarafis P. (2018). Validity and Reliability of the Greek Translation of the Job Satisfaction Survey (JSS). BMC Psychol..

[33] Andreou E., Alexopoulos E.C., Lionis C., Varvogli L., Gnardellis C., Chrousos G.P., Darviri C. (2011). Perceived Stress Scale: Reliability and Validity Study in Greece. Int. J. Environ. Res. Public Health.

[34] Galanis P. (2013). Validity and reliability of questionnaires in epidemiological studies. Arch. Hell. Med..

[35] Hair J.F., Black W.C., Babin B.J., Anderson R.E. (2010). Multivariate Data Analysis.

[36] Papanicolaou G. (2013). Investigation of Job Satisfaction and Stress of Pharmacists Working at the Public Healthcare Sector in Cyprus. Master’s Thesis.

[37] Yeh Y.C., Lin B.Y.J., Lin W.H., Wan T.T.H. (2009). Job Stress: Its Relationship to Hospital Pharmacists’ Insomnia and Work Outcomes. Int. J. Behav. Med..

[38] Carvajal M.J., Popovici I., Hardigan P.C. (2019). Gender and Age Variations in Pharmacists’ Job Satisfaction in the United States. Pharmaceuticals.

[39] Krantz G., Berntsson L., Lundberg U. (2005). Total workload, work stress and perceived symptoms in Swedish male and female white-collar employees. Eur. J. Pub. Health.

[40] Berntsson L., Lundberg U., Krantz G. (2006). Gender differences in work-home interplay and symptom perception among Swedish white-collar employees. J. Epidemiol. Community Health.

[41] Krantz G., Lundberg U. (2006). Workload, work stress, and sickness absence in Swedish male and female white-collar employees. Scand. J. Soc. Health.

[42] Carvajal M.J., Popovici I., Hardigan P.C. (2018). Gender differences in the measurement of pharmacists’ job satisfaction. Hum. Resour. Health.

[43] Lin B.Y.J., Yeh Y.C., Lin W.H. (2007). The Influence of Job Characteristics on Job Outcomes of Pharmacists in Hospital, Clinic, and Community Pharmacies. J. Med. Syst..

[44] Munger M.A., Gordon E., Hartman J., Vincent K., Feehan M. (2013). Community pharmacists’ occupational satisfaction and stress: A profession in jeopardy?. J. Am. Pharm. Assoc..

[45] Khalidi D.A., Wazaify M. (2013). Assessment of pharmacists’ job satisfaction and job related stress in Amman. Int. J. Clin. Pharm..

[46] Teong W.W., Ng Y.K., Paraidathathu T., Chong W.W. (2019). Job satisfaction and stress levels among community pharmacists in Malaysia. J. Pharm. Pract. Res..

[47] Liu C.S., White L. (2011). Key determinants of hospital pharmacy staff’s job satisfaction. Res. Soc. Adm. Pharm..

[48] Manan M.M., Azmi Y., Lim Z., Neoh C.F., Khan T.M., Ming L.C. (2015). Predictors of job satisfaction amongst pharmacists in Malaysian public hospitals and healthcare clinics. J. Pharm. Pr. Res..

[49] Gustafsson M., Mattsson S., Wallman A., Gallego G. (2018). Pharmacists’ satisfaction with their work: Analysis of an alumni survey. Res. Soc. Adm. Pharm..

[50] Mott D.A., Doucette W.R., Gaither C.A., Pedersen C.A., Schommer J.C. (2004). Pharmacists’ Attitudes toward Worklife: Results from a National Survey of Pharmacists. J. Am. Pharm. Assoc..

[51] Gidman W.K., Hassell K., Day J., Payne K. (2007). The impact of increasing workloads and role expansion on female community pharmacists in the United Kingdom. Res. Soc. Adm. Pharm..

[52] Jacobs S., Johnson S., Hassell K. (2018). Managing workplace stress in community pharmacy organisations: Lessons from a review of the wider stress management and prevention literature. Int. J. Pharm. Pract..

[53] Oliveira I.V., Nascimento Y.d.A., Ramalho-de-Oliveira D. (2020). Decision-Making Process in Comprehensive Medication Management Services: From the Understanding to the Development of a Theoretical Model. Pharmacy.

